# Model-Reconstructed RBFNN-DOB for FJR Trajectory Control with External Disturbances

**DOI:** 10.3390/s25185608

**Published:** 2025-09-09

**Authors:** Tianmeng Li, Caiwen Ma, Yanbing Liang, Fan Wang, Zhou Ji

**Affiliations:** 1 Xi’an Institute of Optics and Precision Mechanics, Chinese Academy of Sciences, Xi’an 710119, China; litianmeng@opt.ac.cn (T.L.); cwma@opt.ac.cn (C.M.); lyb@opt.ac.cn (Y.L.); jizhou20@mails.ucas.ac.cn (Z.J.); 2 University of Chinese Academy of Sciences, Beijing 100049, China; 3 Key Laboratory of Space Precision Measurement Technology, Chinese Academy of Sciences, Xi’an 710119, China

**Keywords:** radial basis function neural networks (RBFNN), disturbance observer (DOB), flex joint robot (FJR), nonlinear flexible dynamics, disturbance compensation

## Abstract

Parameter uncertainties and fluctuating disturbances have posed significant challenges to the smooth and precise control of Flexible Joint Robots (FJRs) in industrial environments. To mitigate such disturbances, Disturbance Observers (DOBs) are commonly employed; however, the model uncertainties inherent in FJR systems make accurate dynamic modeling challenging, and the efficacy of DOBs hinges heavily on the accuracy of the dynamic model, which limits their applicability to FJR control. This paper presents a hybrid RBFNN-based Disturbance Observer (RBFNNDOB) state feedback controller for FJRs. By combining a nominal model-based DOB with an RBFNN, this method effectively addresses the unknown dynamics of FJRs while simultaneously compensating for external time-varying disturbances. In this framework, an adaptive neural network weight update law is formulated using Lyapunov stability theory. This enables the RBFNN to selectively estimate the unmodeled uncertainties in FJR dynamics, thereby minimizing computational redundancy in model estimation while allowing dynamic compensation for residual uncertainties beyond the nominal model and DOB estimation errors—ultimately enhancing computational efficiency and achieving robust compensation for rapidly changing disturbances. The boundedness of the tracking error is proven using the Lyapunov approach, and experimental validation is conducted on the FJR system to confirm the efficacy of the proposed control method.

## 1. Introduction

Flexible Joint Robots (FJRs) are more robust, energy-efficient, and safer for human-robot interaction than rigid-joint robotic arms. This makes them useful in many areas, including healthcare, aerospace, and industrial automation. Common uses include variable-stiffness actuators for rehabilitation prosthetics and lifting heavy objects [1,2,3], as well as space robots that maintain satellites and process debris [4,5,6].

Flexible joints naturally create uncertainties in the dynamic models of robotic systems [7,8], making it hard for traditional rigid-body tracking control methods to work well [9]. When modeling the dynamics of flexible joints, a lumped-parameter approach usually describes the connection between the link and rotor using a linear torsional spring with stiffness coefficient *k*. This elastic link changes the system’s Lagrangian structure in a basic way, resulting in an underactuated setup where the dynamic order is twice that of rigid-body systems. The resulting non-minimum phase dynamics—where the number of state-space dimensions is greater than the number of control inputs—put built-in limits on how well trajectory tracking can perform. What’s more, under the same disturbance conditions, robots with flexible joints vibrate more than those with rigid joints, and this significantly hurts the controller’s performance.

The presence of flexible joints in robotic systems invalidates conventional control strategies designed for rigid manipulators, necessitating the development of novel controllers tailored to FJRs. The PD controller can stabilize FJRs without requiring an accurate dynamic model [10]. To address flexible vibrations in FJRs and improve closed-loop stability, researchers have implemented state observers to estimate system uncertainties and external disturbances, with real-time compensation based on these estimates. Among such methods, Disturbance Observers (DOBs) stand out as one of the most widely adopted approaches. Typically, DOBs can be combined with PD controllers to enhance the robustness of the control system. Kim et al. [11] utilized DOB for motor-side disturbance estimation alongside PD stabilization, while Lee et al. [12] designed a novel Rotating Workspace Nonlinear Disturbance Observer (RWNDO) to eliminate both disturbances and couplings of the FJR in the rotating workspace. However, inherent mismatches between nominal and actual system models may compromise the stability of DOB control architectures [13].

As intelligent control algorithms advance, researchers have started incorporating them into controllers for FJRs. Radial Basis Function (RBF) neural networks, with their ability to approximate continuous functions universally when sufficiently configured, effectively handle nonlinearities in real-world FJR systems—these stem from distributed compliance sources like gear deformation, belt/tendon flexibility, and hydraulic dynamics that don’t fit the ideal linear spring model—and also address unmeasurable nonlinear factors such as time-varying load disturbances, nonsmooth friction, and sensor quantization errors. The inherent uncertainty of FJRs makes RBF neural networks a good choice for real-time estimation and compensation of unmodeled dynamics in flexible-joint manipulators [14,15], which not only eases the workload of controller design but also effectively estimates unknown system components and boosts controller accuracy. Importantly, RBF neural networks are robust to input noise and small data changes, helping enhance the control system’s anti-interference capability. To achieve precise trajectory tracking control for flexible-joint manipulators under complex constraints, Pan et al. [16] proposed a new neuro-adaptive command-filtered output-feedback backstepping control scheme that mitigates input saturation, estimates unmeasurable states, and eliminates complexity explosion. However, their gradient-based weight adjustment leads to unavoidable computational delays that cause phase mismatches when disturbances change suddenly, potentially hurting performance in high-bandwidth tracking applications [17].

In recent years, researchers have aimed to address DOB approximation errors arising from model uncertainties and the limitations of RBF neural networks in compensating for fast-changing disturbances by proposing control methods that combine neural networks with DOBs. Phan et al. [18] proposed an adaptive neural network to compensate for manipulator dynamics uncertainties and designed a disturbance observer to suppress non-parametric disturbances. Zhang et al. [19] proposed a novel adaptive neural network control method based on a disturbance observer for robotic systems with variable stiffness joints and affected by model uncertainties. Ma et al. [20] proposed an adaptive sliding mode control strategy for lower limb rehabilitative robots, integrating disturbance observers for bounded disturbance rejection and neural networks for uncertainty approximation (with a chattering-reducing switching function). Xu et al. [21] used a neural network to estimate the model for a double-inertia model with double gear meshing, employed PD control to stabilize the system, and utilized a DOB to estimate and compensate for unknown nonlinear disturbances. Yan et al. [22] present a time-varying disturbance-observer-based improved sliding-mode control (TVDO-ISMC) method for tracking control of uncertain flexible-joint manipulators (UFJMs), which establishes an error system, estimates disturbances via TVDO, overcomes uncertainties, and reduces chattering. While the above methods effectively reduce the DOB’s reliance on precise system models, they replace the required model information with neural network estimates during design. This neural network estimation of unknown dynamics imposes significant computational load, inherently degrading controller response speed.

To reduce computational complexity while addressing parametric uncertainties in FJR systems and improving both tracking accuracy and dynamic response characteristics, this paper proposes an RBFNN-based DOB state feedback controller. The method utilizes the FJR nominal model to design the state feedback controller and DOB. Building on this foundation, a reconstruction model based on the FJR nominal model and the observer’s estimated values is proposed. A novel weight update law is designed for the RBFNN, reducing computational redundancy in FJR model estimation while enabling dynamic compensation for residual uncertainties beyond the FJR nominal model and DOB estimation errors. This mechanism ensures robust tracking performance under complex dynamic conditions.

The main contributions of this paper are listed below:

(1) We combine RBFNN with DOB to suppress external disturbances and compensate for uncertain parameters in the FJR system. Specifically, DOB can estimate external disturbances based on the nominal model; however, traditional DOB may introduce errors in the estimated values due to the model’s uncertain parameters, and in extreme cases, even cause system instability. Conversely, RBFNN is employed to compensate for the influence of uncertain parameters beyond the FJR’s nominal model, and to correct errors in the disturbance estimates from the DOB. Their integration enhances control accuracy, mitigates interference effects, and significantly accelerates error convergence, thereby achieving superior performance characterized by higher precision and faster response.
(2) This paper proposes a new Lyapunov-function-based adaptive update law for RBFNN weights. A key advantage lies in its significantly reduced computational burden compared to existing methods: unlike the approaches in [18,19,21] that employ RBFNN to estimate the entire model (incurring heavy computational loads due to the need for full-model approximation), our method uses the discrepancy between the FJR’s actual output and the state reconstruction model’s output (based on DOB and RBFNN estimates) as the RBFNN input. This streamlined input design, combined with the proposed adaptive law, not only enables direct estimation of unknown parameters but also substantially cuts down on computational complexity. The reduced computational requirements directly enhance RBFNN convergence efficiency and minimize errors, making the approach more suitable for real-time control applications.
(3) In instances where the unknown parameters of the FJR model or disturbances are minimal or non-existent—i.e., when state reconstruction model errors are minimal or absent—the RBFNN in the controller requires little to no compensation for the system. For cases with large state reconstruction model errors, the RBFNN compensates for the system. Compared with controllers using only DOB, the controller proposed in this paper achieves superior error suppression performance.
(4) Through simulations and experiments, stable trajectory tracking of the FJR is demonstrated under conditions of external disturbances or structural self-changes, which validates the feasibility and superiority of the proposed method. Specifically, compared with traditional methods, the proposed method achieves a 47.32% reduction in RMSE for disturbance suppression and a 63.47% reduction in RMSE for trajectory tracking. Moreover, it remains effective even when the inertia of the model is changed, further confirming its robustness.

A stability analysis of the closed-loop system is presented based on the Lyapunov method. The effectiveness of the proposed control is analyzed via simulation and verified experimentally in a real-time system.

The rest of this article is organized as follows. In Section 2, the dynamical model of the underactuated FJR system with its coordinate transformations is presented. The control law and the design of the stability proof are given in Section 3. In Section 4, the simulation and experimental results are provided to illustrate the effectiveness of the proposed control approach. Finally, Section 5 concludes this article.

## 2. System Dynamic Modeling

In this section, the research object of this paper is the rotary flexible joint manipulator manufactured by Quanser. The dynamic model of the flexible-joint robot arm is presented. To simplify controller design, a coordinate transformation is performed on the manipulator dynamic model.

### 2.1. Description of the Single-Link FJR

**Figure 1:**
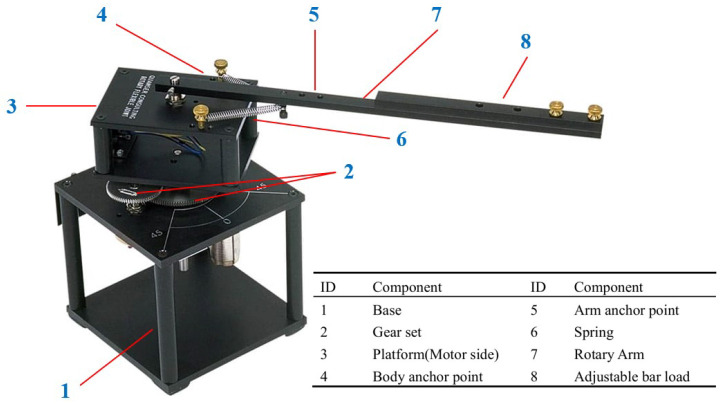
Experimental platform of an FJR system.

Figure 1 shows a rotary flexible-joint manipulator manufactured by Quanser, consisting of a DC motor, an aluminum frame, two encoders, a freely moving rigid link, a high-gear assembly, and a pair of identical springs. The DC motor delivers driving torque to the link through the gear and springs. The flexibility of these two springs causes oscillations, which can significantly impact the movement of the link.

**Figure 2:**
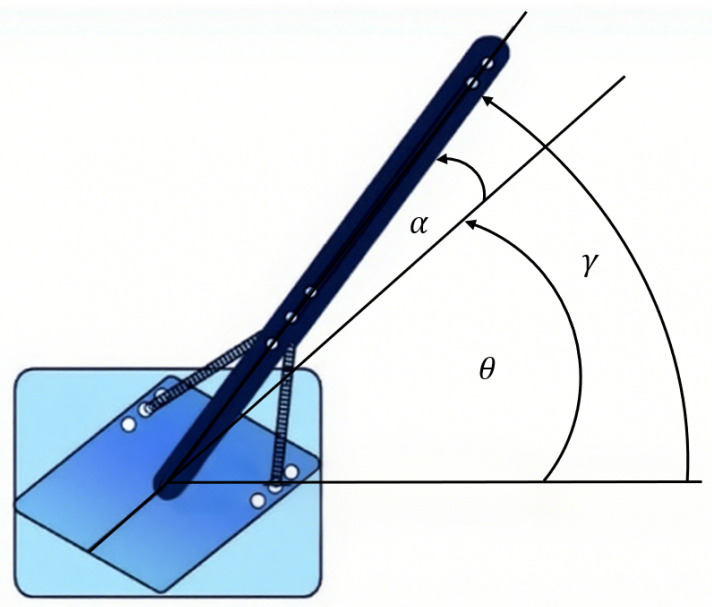
The angular position of the FJR system.

As depicted in Figure 2, θ and α represent the angular position of the motor and the angular deflection of the link, measured by the encoder at the motor end and the encoder at the link base, respectively. Furthermore, γ is the total motion of the end-effector of the link, which is the sum of θ and α.

### 2.2. FJR Dynamics Modeling

The FJR dynamics can be described by the link dynamics and the motor dynamics as follows [23]:

(1) $$\begin{array}{cc} \ddot{\alpha }\left(t\right)-a_{1}\alpha \left(t\right)-a_{3}\dot{\theta }\left(t\right) & =b_{1}u\left(t\right) \end{array}$$

(2) $$\begin{array}{cc} \ddot{\theta }\left(t\right)-a_{2}\alpha \left(t\right)-a_{4}\dot{\theta }\left(t\right) & =b_{2}u\left(t\right) \end{array}$$

where $b_{1}=-\frac{\eta_{m}\eta_{g}K_{t}K_{g}}{J_{\mathrm{eq}}R_{m}}$, $b_{2}=-b_{1}$, $a_{1}=-\frac{K_{s}\left(J_{\mathrm{eq}}+J_{\mathrm{arm}}\right)}{J_{\mathrm{eq}}J_{\mathrm{arm}}}$, $a_{2}=\frac{K_{s}}{J_{\mathrm{eq}}}$, $a_{3}=\frac{\eta_{m}\eta_{g}K_{t}K_{m}K_{g}^{2}+B_{\mathrm{eq}}R_{m}}{J_{\mathrm{eq}}R_{m}}$, and $a_{4}=-a_{3}$. The input *u* is the voltage applied to the motor. Here, $J_{\mathrm{arm}}$, $J_{\mathrm{eq}}$, $B_{\mathrm{eq}}$, and $K_{s}$ denote the nominal plant parameters listed in Table 1.

Based on Table 1, we can derive the dynamic model of the FJR. However, because of factors like sensor errors, nonlinearities in flexible joints, and system noise, there are unavoidable differences between the actual FJR parameters and those listed in Table 1. This means it’s impossible to get a fully accurate dynamic model. To address these errors, this paper takes into account the impact of the FJR’s unmodeled components on the system, which are denoted as Δ.

Based on (1) and (2) with considering uncertainties, external disturbances, and nonlinearities caused by the flexibility, the state-space form is described as

(3) $$\begin{array}{cc} \; & {\dot{\eta }}_{1}=\eta_{2}\\ \; & {\dot{\eta }}_{2}=f_{10}\left(\alpha ,\dot{\theta }\right)+b_{10}u+d_{1}+\Delta_{1}\\ \; & {\dot{\eta }}_{3}=\eta_{4}\\ \; & {\dot{\eta }}_{4}=f_{20}\left(\alpha ,\dot{\theta }\right)+b_{20}u+d_{2}+\Delta_{2} \end{array}$$

where $\eta ={\left[\begin{array}{cccc} \eta_{1} & \eta_{2} & \eta_{3} & \eta_{4} \end{array}\right]}^{T}={\left[\begin{array}{cccc} \alpha & \dot{\alpha } & \theta & \dot{\theta } \end{array}\right]}^{T}$ represents a vector consisting of state variables of the system, $b_{10}$ and $b_{20}$ are nominal components of $b_{1}$ and $b_{2}$, respectively, $f_{10}(\alpha ,\dot{\theta })$ and $f_{20}(\alpha ,\dot{\theta })$ are smooth linear functions in terms of systems states for the link and the motor, respectively, which are described as

(4) $$\begin{array}{cc} \; & f_{10}\left(\alpha ,\dot{\theta }\right)=a_{1}\alpha \left(t\right)+a_{3}\dot{\theta }\left(t\right)\\ \; & f_{20}\left(\alpha ,\dot{\theta }\right)=a_{2}\alpha \left(t\right)+a_{4}\dot{\theta }\left(t\right) \end{array}$$

Furthermore, $d_{1}$ and $d_{2}$ represent external nonlinear disturbances, such as unintentional human contact or collisions with objects, which may cause unexpected displacements of the FJR. $\Delta_{1}$ and $\Delta_{2}$ represent the errors between the nominal dynamic model and the actual dynamic model. These errors are defined as follows:

(5) $$\begin{array}{cc} \; & \Delta_{1}=f_{1}\left(\alpha ,\dot{\theta }\right)-f_{10}\left(\alpha ,\dot{\theta }\right)+b_{1}u-b_{10}u\\ \; & \Delta_{2}=f_{2}\left(\alpha ,\dot{\theta }\right)-f_{20}\left(\alpha ,\dot{\theta }\right)+b_{2}u-b_{20}u \end{array}$$

where $f_{1}\left(\alpha ,\dot{\theta }\right)$, $f_{2}\left(\alpha ,\dot{\theta }\right)$; $b_{1}$, $b_{2}$ represent the parameters of the dynamic equation of the real-world FJR. The forms and parameter dependencies of $f_{1}\left(\alpha ,\dot{\theta }\right)$ and $f_{2}\left(\alpha ,\dot{\theta }\right)$ in Equation (5) stem from the dynamic characteristics of the Rotary Flexible Joint system, as detailed in [23].

It can be seen that there are errors between the nominal dynamic model of the FJR and the actual dynamic model that need to be resolved. The elimination of errors and disturbances will be described in Section 3.

### 2.3. Coordinate Transformation of the FJR Manipulator

In Section 2.2, we use θ and α to represent the system’s dynamic model, as shown in (3). From the earlier analysis, we can see that the system contains mismatched disturbances. To simplify controller design, we apply a coordinate transformation to the FJR’s dynamic Equation (3), which further converts the system’s external disturbance term into a matched disturbance—this facilitates subsequent controller design.

We define the following new states by utilizing the Olfati transformation [24]:

(6) $$\begin{array}{cc} \; & z_{1}=\eta_{1}-\int_{0}^{\eta_{3}}\frac{b_{10}\left(s\right)}{b_{20}\left(s\right)}\;ds=\eta_{1}+\eta_{3}\\ \; & z_{2}=\eta_{2}-\frac{b_{10}\left(\eta \right)}{b_{20}\left(\eta \right)}\eta_{4}=\eta_{2}+\eta_{4}\\ \; & z_{3}=\eta_{3}\\ \; & z_{4}=\eta_{4} \end{array}$$

where $b_{10}=-\frac{\eta_{m}\eta_{g}K_{t}K_{g}}{J_{eq0}R_{m}}$, $b_{20}=-b_{10}$.

Taking the time derivative of (6) and substituting the dynamics (3), the system (3) becomes:

(7) $$\begin{array}{cc} \; & {\dot{z}}_{1}=z_{2}\\ \; & {\dot{z}}_{2}=z_{3}+d_{1}\left(x,w_{1},w_{2}\right)\\ \; & {\dot{z}}_{3}=z_{4}\\ \; & {\dot{z}}_{4}=f_{20}\left(\alpha ,\dot{\theta }\right)+b_{20}u+d_{2}+\Delta_{2} \end{array}$$

where $z={\left[z_{1},z_{2},z_{3},z_{4}\right]}^{T}={\left[\gamma ,\dot{\gamma },\theta ,\dot{\theta }\right]}^{T}$ and $d_{1}\left(x,w_{1},w_{2}\right)=-z_{3}+f_{10}+f_{20}+d_{1}+d_{2}+\Delta_{1}+\Delta_{2}$.

**Remark** **1.** *Note that the Olfati transformation serves to convert the original FJR system (3) into a normal strict form (7). Like many robotic systems, the FJR system belongs to Class-I underactuated systems [25], which include the inertia wheel pendulum, rotary inverted pendulum, and others. This classification arises because the control coefficients $b_{10}$ and $b_{20}$ are known nonzero constants, indicating that the moment.*

Although the underactuation in (3) is removed, the mismatched disturbance appears in (7). In order to transfer the mismatched to matched disturbance in (7), we apply a flatness approach for this system dynamics by differentiating its output $z_{1}\left(t\right)$ four times till the input–output relation is obtained since the relative degree of the system is 4. Defining new coordinates as $x_{1}\left(t\right)=z_{1}\left(t\right)$, $x_{2}\left(t\right)={\dot{z}}_{1}\left(t\right)$, $x_{3}\left(t\right)={\ddot{z}}_{1}\left(t\right)$ and $x_{4}\left(t\right)={\dddot{z}}_{1}\left(t\right)$ the dynamics (7) can be written in terms of the new states as:

(8) $$\begin{array}{cc} \; & {\dot{x}}_{1}=x_{2}\\ \; & {\dot{x}}_{2}=x_{3}\\ \; & {\dot{x}}_{3}=x_{4}\\ \; & {\dot{x}}_{4}=f_{0}\left(x\right)+g\left(x\right)u+d+\Delta \end{array}$$

where $x={\left[\begin{array}{cccc} x_{1} & x_{2} & x_{3} & x_{4} \end{array}\right]}^{T}={\left[\begin{array}{cccc} \gamma & \dot{\gamma } & \ddot{\gamma } & \dddot{\gamma } \end{array}\right]}^{T}$ represent the position, velocity, acceleration, and time derivative of the acceleration of the link, respectively. According to (1), (2) and (3), the functions can be derived as $f_{0}\left(x\right)=\left(a_{1}+a_{2}\right)f_{10}\left(\alpha ,\dot{\theta }\right)$ and $g\left(x\right)=\left(a_{1}+a_{2}\right)b_{10}u$. And $d=\left(a_{1}+a_{2}\right)d_{1}$ represents the total external disturbance acting on the connecting rod. Δ represents the unknown part of the system beyond the FJR nominal model, including system dynamics and unmodelled dynamics. It is expressed as:

(9) $$\Delta =f\left(x\right)-f_{0}\left(x\right)$$

where f(x) denotes the actual state of the JFR system, it remains unknown and hard to acquire through measurements. This corresponds to $f_{0}\left(x\right)$, the state of the nominal model.

For the dynamic model (8) of the FJR resulting from the coordinate transformation, the control objectives can be categorized into the following three points. (a) We need to ensure that the motion of the end effector is consistent with its desired position. This is the primary objective of the control system ($x_{1}=\gamma_{d}$). (b) The control system needs to make the angular position of the motor θ follow its desired trajectory to ensure that the motion of the FJR meets the expected requirements. (c) We need to minimize the angular deflection of the link α through the control system to improve the stability and accuracy of the system. Therefore, it is very important to design appropriate controller to achieve these objectives.

**Remark** **2.** *The two parameters $x_{3}$ and $x_{4}$ correspond to acceleration and the derivative of acceleration, respectively. These parameters are difficult to measure directly with an encoder but can be calculated via mathematical transformation from the measured parameter α. As noted in Section 2 (see (9)), the research object has a complex model, making it impossible to derive an accurate mathematical model of the controlled system. Additionally, real-world systems involve total disturbances, including system dynamics, unmodeled dynamics, and external disturbances. While the disturbance observer proposed in Section 3.2 can estimate part of the external disturbance, this chapter focuses on studying the error *Δ* between the actual system model and the nominal model.*

## 3. Controller Design and Stability Analysis

To achieve the above objectives, this section introduces a controller based on the Radial Basis Function Neural Network-Disturbance Observer (RBFNN-DOB). The DOB, which relies on the nominal model, cannot accurately estimate disturbances—this could potentially destabilize the FJR system. To address this issue, an RBFNN is used to compensate for the system’s unknown components, including system dynamics, unmodeled dynamics, and the partial disturbance estimation errors from the DOB. Additionally, the neural network’s update rate is determined using the Lyapunov method.

### 3.1. State Feedback Controller Design

It was demonstrated by Tomei in 1991 [10] that a simple PD controller can be used to control a flexible-joint robotic arm. I. H. Akyuz [26] proved by experiments that state feedback control and PID control can achieve similar control accuracy. Therefore, for the plant (8) proposed in Section 2, a corresponding state feedback controller can be designed, and the state parameter can be changed to the error between the output and the target value according to the algorithm proposed in this paper:

(10) $$u=\frac{1}{g(x)}\left[-f_{0}\left(x\right)-\hat{d}-\Delta +K^{T}E+{\ddot{x}}_{3d}\right]$$

where *K* is the designed state feedback control parameter $K={\left[\begin{array}{cccc} Kp_{1} & Kd_{1} & Kp_{2} & Kd_{2} \end{array}\right]}^{T}$, and $E={\left[\begin{array}{cccc} e_{1} & e_{2} & e_{3} & e_{4} \end{array}\right]}^{T}=x_{d}-x$ represents the concatenated error states of the FJR system, comprising position error $\gamma_{d}-\gamma$, velocity error ${\dot{\gamma }}_{d}-\dot{\gamma }$, acceleration error ${\ddot{\gamma }}_{d}-\ddot{\gamma }$, and jerk (third derivative) error ${\dddot{\gamma }}_{d}-\dddot{\gamma }$. It is worth noting that the state feedback controller is a performance controller for the system and is responsible for improving the performance of the system. DOB also needs to be added to ensure the robustness of the system. The two-degree-of-freedom control structure of the robust control system based on DOB was proposed in [21]. Experiments have shown that the robustness and performance of the control system can be independently adjusted by DOB and the performance controller, respectively [21].

Given the inherent complexity of FJR systems, deriving an accurate dynamic model remains infeasible, necessitating the retention of two unknown parameters-*d* and Δ-within the controller architecture. These quantities, defined in Section 3.2 and Section 3.3 respectively, are explicitly introduced to account for unmodeled dynamics and approximation errors. Their computational procedures are systematically elaborated in the subsequent subsections to ensure traceability and reproducibility.

### 3.2. DOB Design

Due to the physical characteristics of the FJR itself, even small disturbances can lead to system performance being affected or even instability. As can be seen in the system (8), the FJR is affected by the disturbance *d*, and an artificially estimated $\hat{d}$ needs to be added to the control law to cancel out external disturbances.

The concept of DOB was introduced in [27] and has since become a widely adopted robust control technique for addressing model uncertainties and external disturbances in controller design. In this section, a corresponding disturbance observer can be designed to achieve disturbance estimation.

**Assumption** **1.** *The external disturbance d(t) is assumed to be bounded, namely there exists an unknown positive constant $d_{m}$ making $d\left(t\right)\leq d_{m}$.*

Firstly, we define as the estimation of unknown disturbance $\hat{d}$, with

(11) $$\hat{d}+\tilde{d}=d$$

where $\tilde{d}$ is the estimation error.

Secondly, Applying RBFNN to approximate the rest unknown dynamics uncertainties, we define

(12) $$\Delta +\tilde{d}=W^{T}h\left(x\right)$$

where *W* is the NN weight and h(x) is the radial basis function output, The detailed description of the RBFNN is in the next Section 3.3.

Theoretically RBFNN has the ability to smoothly approximate all continuous function. However, here in practice, errors always occur during updating weight, so we define it to be

(13) $$\tilde{W}=\hat{W}-W^{*}$$

where $W^{*}$ denotes the ideal updating weight, and $\hat{W}$ represents the practical estimate of this ideal updating weight.

To complete the formulation of DOB, an auxiliary equation is designed as

(14) z=d−p(x)

where p(x) is the nonlinear function to be designed—a custom-built core component of the disturbance observer. In this work, p(x) is constructed to satisfy $\dot{p}\left(x\right)=l\left(x\right)\dot{x}$, forming the basis of disturbance estimation while determining the observer gain through its derivative, thus embodying a tailored design logic for nonlinear systems. The nonlinear disturbance observer gain l(x) is determined by

(15) $$l\left(x\right)=\frac{\partial p(x)}{\partial x}$$

Considering (14), we calculate the derivative of *z* regarding to time

(16) $$\dot{z}=\dot{d}-l\left(x\right)\left[f_{0}\left(x\right)+g\left(x\right)u+d+W^{T}h\left(x\right)\right]$$

Combining Assumption 1 we assume that the system disturbance is slowly time varying.

Thus (16) can be updated to be

(17) $$\dot{\hat{z}}=-l\left(x\right)\left[f_{0}\left(x\right)+g\left(x\right)u+\hat{d}+{\hat{W}}^{T}h\left(x\right)\right]$$

where $\hat{d}=\hat{z}+p\left(x\right)$. Equation (17) can therefore be expressed as

(18) $$\dot{\hat{z}}=-l\left(x\right)\hat{z}-l\left(x\right)\left[p\left(x\right)+f_{0}\left(x\right)+g\left(x\right)u+{\hat{W}}^{T}h\left(x\right)\right]$$

where $x\in R^{n}$, $u\in R^{m}$, $d\in R^{l}$, $y\in R^{s}$ are the state vector, control input vector, disturbance vector, and output vector, respectively.

**Remark** **3.** *In the proposed controller, the Disturbance Observer (DOB) is designed based on a nominal model, treating the controlled system as a gray-box model with partially known information. Although FJR have complex structures, approximate dynamic models can be established by measuring partial data (e.g., inertia, stiffness, friction coefficients). By contrast, References [18,19,20,21] treats the plant as a black-box model with completely unknown information, estimating all model parameters via neural networks. The proposed controller exhibits reduced computational complexity while theoretically achieving higher control accuracy and faster response compared to the black-box approach.*

**Figure 3:**
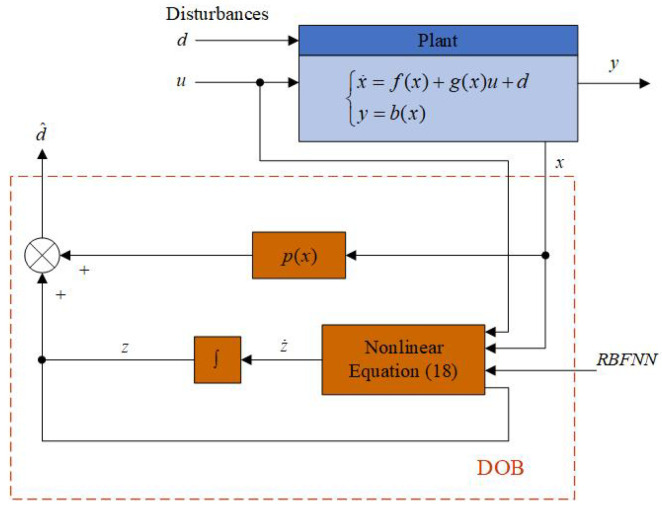
Block diagram of the DOB.

The block diagram of the disturbance observer for system (8) is given by Figure 3.

### 3.3. RBF Neural Network Disturbance Observers Design and Stability Analysis

As noted in Section 2 (see (9)), the research object has a complex model, making it impossible to derive an accurate mathematical model of the controlled system. Additionally, real-world systems involve total disturbances, including system dynamics, unmodeled dynamics, and external disturbances. While the disturbance observer proposed in Section 3.2 can estimate part of the external disturbance, this chapter focuses on studying the error Δ between the actual system model and the nominal model.

RBF neural networks have attracted much attention due to their good generalization ability and simple network structure, which avoids unnecessary and lengthy calculations. According to research, RBFNN can approximate any nonlinear function in a compact set and arbitrary precision [28,29,30]. This subsection will design a corresponding RBFNN to estimate the error Δ.

The general form of an RBF neural network is:

(19) $$\begin{array}{cc} \; & h_{j}=g\left(\frac{{∥x-c_{ij}∥}^{2}}{{b_{j}}^{2}}\right)\\ \; & f=W^{T}h\left(x\right)+\omega \end{array}$$

where *x* is the input to the RBFNN; *i* is the number of inputs to the RBFNN; *j* is the *j*th node of the hidden layer of the RBFNN; $h={\left[h_{1},h_{2},\cdot \cdot \cdot ,h_{n}\right]}^{T}$ is the output of the Gaussian function; *W* is the weight of the RBFNN; and ε is the approximation error of the RBFNN.

**Theorem** **1.** *Define $\hat{\Delta }={\hat{W}}^{T}h$. The $\hat{W}$ adaptive algorithm is designed to*

(20) $$\dot{\hat{W}}=-\mu \left(E^{T}-{\hat{E}}^{T}\right)PBh\left(x\right)+\mu \delta \hat{W}$$

*where $\hat{e}=z_{d}-\hat{z}$, $\hat{E}={\left[\begin{array}{cccc} {\hat{e}}_{1} & {\hat{e}}_{2} & {\hat{e}}_{3} & {\hat{e}}_{4} \end{array}\right]}^{T}$ is the error between the actual outputs of the FJR and its estimated state outputs.*

**Proof of Theorem** **1.** Substituting the control law (10) into system (8) yields:

(21) $${\dot{e}}_{4}=K^{T}E-\left(d-\hat{d}\right)+\left({\hat{\Delta }}_{2}-\Delta_{2}\right)$$

Define: $\Lambda =\left[\begin{array}{cccc} 0 & 1 & 0 & 0\\ 0 & 0 & 1 & 0\\ 0 & 0 & 0 & 1\\ -Kp_{1} & -Kd_{1} & -Kp_{2} & -Kd_{2} \end{array}\right]$, $B={\left[\begin{array}{cccc} 0 & 0 & 0 & 1 \end{array}\right]}^{\;T}$.

The optimal weight values of the RBFNN can be written as:

(22) $$W^{*}=\arg\min_{W\in \Omega }\;\left[\sup\left|\left(\hat{\Delta }-\Delta \right)\right|\right]$$

The model approximation error is defined as:

(23) $$\omega =\hat{\Delta }\left(x\left|W^{*}\right.\right)-\Delta$$

Since ∥B∥=1 and thus the approximation error of the neural network is bounded, we obtain $\left|\omega \right|\leq \omega_{N}$.

Then the closed-loop system can be written as:

(24) $$\dot{E}=\Lambda E+B\left[-\tilde{d}+{\left(\hat{W}-W^{*}\right)}^{T}h\left(x\right)+\omega \right]$$

The Lyapunov function is designed as:

(25) $$V=\frac{1}{2}E^{T}PE+\frac{1}{2\mu }{\left(\hat{W}-W^{*}\right)}^{T}\left(\hat{W}-W^{*}\right)+\frac{1}{2}{\hat{E}}^{T}P\hat{E}+\frac{1}{2}{\tilde{d}}^{2}$$

Among them, μ is a normal number, the matrix is symmetric and positive definite, and satisfies the following Lyapunov equation:

(26) $$\Lambda^{T}P+P\Lambda =-Q$$

where Q>0.

Define $V_{1}=\frac{1}{2}E^{T}PE$, $V_{2}=\frac{1}{2\mu }{\left(\hat{W}-W^{*}\right)}^{T}\left(\hat{W}-W^{*}\right)$, $V_{3}=\frac{1}{2}{\hat{E}}^{T}P\hat{E}$, $V_{4}=\frac{1}{2}{\tilde{d}}^{2}$ and $M=B[-\tilde{d}+{\left(\hat{W}-W^{*}\right)}^{T}h\left(x\right)+\omega ]$. Then the closed-loop Equation (24) can be written as:

(27) $$\dot{E}=\Lambda E+M$$

Then:

(28) $$\begin{array}{cc} {\dot{V}}_{1} & =\frac{1}{2}{\dot{E}}^{T}PE+\frac{1}{2}E^{T}P\dot{E}\\ \; & =\frac{1}{2}\left(E^{T}\Lambda^{T}+M^{T}\right)PE+\frac{1}{2}E^{T}P\left(\Lambda E+M\right)\\ \; & =\frac{1}{2}E^{T}\left(\Lambda^{T}P+P\Lambda \right)E+\frac{1}{2}M^{T}PE+\frac{1}{2}E^{T}PM\\ \; & =-\frac{1}{2}E^{T}QE+\frac{1}{2}\left(M^{T}PE+E^{T}PM\right)\\ \; & =-\frac{1}{2}E^{T}QE+E^{T}PM \end{array}$$

Derivative of $V_{3}$, then

(29) $$\begin{array}{cc} \; & {\dot{V}}_{3}=\frac{1}{2}{\dot{\hat{E}}}^{T}P\hat{E}+\frac{1}{2}{\hat{E}}^{T}P\dot{\hat{E}}\\ \; & ={\hat{E}}^{T}P\left[\tilde{d}-{\left(\hat{W}-W^{*}\right)}^{T}h\left(x\right)-\omega -BB^{T}P^{T}\hat{E}\right] \end{array}$$

Derivative of $V_{4}$, then:

(30) $$\begin{array}{cc} {\dot{V}}_{4} & =\tilde{D}\dot{\tilde{D}}\\ \; & =\tilde{d}\dot{d}-\tilde{d}l\left(x\right)\left[\tilde{d}-{\left(\hat{W}-W^{*}\right)}^{T}h\left(x\right)\right]\\ \; & =\tilde{d}\dot{d}-l\left(x\right){\tilde{d}}^{2}+\tilde{d}l\left(x\right){\left(\hat{W}-W^{*}\right)}^{T}h\left(x\right) \end{array}$$

Substituting *M* into (28) and (29), because of $E^{T}PB{\left(\hat{W}-W^{*}\right)}^{T}h\left(x\right)=$${\left(\hat{W}-W^{*}\right)}^{T}\left[E^{T}PBh\left(x\right)\right]$, then

(31) $$\begin{array}{cc} {\dot{V}}_{1} & =-\frac{1}{2}E^{T}QE+E^{T}PB{\left(\hat{W}-W^{*}\right)}^{T}h\left(x\right)+E^{T}PB\left(-\tilde{d}+\omega \right)\\ \; & =-\frac{1}{2}E^{T}QE+{\left(\hat{W}-W^{*}\right)}^{T}E^{T}PBh\left(x\right)+E^{T}PB\left(-\tilde{d}+\omega \right) \end{array}$$

(32) $$\begin{array}{cc} {\dot{V}}_{3} & =-{\hat{E}}^{T}PB{\left(\hat{W}-W^{*}\right)}^{T}h\left(x\right)+{\hat{E}}^{T}PB\left(\tilde{d}-\omega \right)\\ \; & =-{\left(\hat{W}-W^{*}\right)}^{T}{\hat{E}}^{T}PBh\left(x\right)+{\hat{E}}^{T}PB\left(\tilde{d}-\omega \right)-{\hat{E}}^{T}\left(PB\right){\left(PB\right)}^{T}\hat{E} \end{array}$$

(33) $${\dot{V}}_{2}=\frac{1}{\mu }{\left(\hat{W}-W^{*}\right)}^{T}\dot{\hat{W}}$$

Derivative of *V*, then

(34) $$\begin{array}{cc} \dot{V} & ={\dot{V}}_{1}+{\dot{V}}_{2}+{\dot{V}}_{3}+{\dot{V}}_{4}\\ \; & =-\frac{1}{2}E^{T}QE+\frac{1}{\mu }{\left(\hat{W}-W^{*}\right)}^{T}\left[\dot{\hat{W}}+E^{T}PBh\left(x\right)-{\hat{E}}^{T}PBh\left(x\right)\right]\\ \; & \;-{\hat{E}}^{T}\left(PB\right){\left(PB\right)}^{T}\hat{E}+E^{T}PB\left(-\tilde{d}+\omega \right)+{\hat{E}}^{T}PB\left(\tilde{d}-\omega \right)-l\left(x\right){\tilde{d}}^{2}+\tilde{d}l\left(x\right){\left(\hat{W}-W^{*}\right)}^{T}h\left(x\right) \end{array}$$

Substituting (20) into (34), then

(35) $$\begin{array}{cc} \dot{V} & =-\frac{1}{2}E^{T}QE-{\hat{E}}^{T}\left(PB\right){\left(PB\right)}^{T}\hat{E}-{\left(\hat{W}-W^{*}\right)}^{T}\delta \hat{W}\\ & +E^{T}PB\left(-\tilde{d}+\omega \right)+{\hat{E}}^{T}PB\left(\tilde{d}+\omega \right)-l\left(x\right){\tilde{d}}^{2}\\ & +\tilde{d}l\left(x\right){\left(\hat{W}-W^{*}\right)}^{T}h\left(x\right)\\ \; & =-\frac{1}{2}E^{T}QE-{\hat{E}}^{T}\left(PB\right){\left(PB\right)}^{T}\hat{E}-{\tilde{W}}^{T}\delta \hat{W}\\ & +E^{T}PB\left(-\tilde{d}+\omega \right)+{\hat{E}}^{T}PB\left(\tilde{d}-\omega \right)\\ & -l\left(x\right){\tilde{d}}^{2}+\tilde{d}l\left(x\right){\tilde{W}}^{T}h\left(x\right) \end{array}$$

To further advance the proof, we leverage the Arithmetic-Geometric Mean (AM-GM) Inequality to perform a scaling operation on (35). Through this process, the following formulas are derived:

(36) $$\tilde{d}l\left(x\right)\tilde{W}h\left(x\right)\leq \frac{{\tilde{d}}^{2}}{2}+\frac{l\left(x\right){∥\tilde{W}∥}^{2}h\left(x\right)}{2}$$

(37) $$-{\tilde{W}}^{T}\delta \hat{W}\leq -\delta {∥\tilde{W}∥}^{2}+\frac{1}{2}\delta {∥\tilde{W}∥}^{2}+\frac{1}{2}\delta {∥W^{*}∥}^{2}$$

(38) $$-E^{T}PB\tilde{d}\leq \frac{1}{2}E^{T}\left(PB\right){\left(PB\right)}^{T}E+\frac{1}{2}{\tilde{d}}^{2}$$

(39) $${\hat{E}}^{T}PB\tilde{d}\leq \frac{1}{2}{\hat{E}}^{T}\left(PB\right){\left(PB\right)}^{T}\hat{E}+\frac{1}{2}{\tilde{d}}^{2}$$

Using the scaling operations described prior, we rewrite the Lyapunov derivative (35) as:

(40) $$\begin{array}{cc} \dot{V}\leq & -\frac{1}{2}E^{T}QE+\frac{1}{2}E^{T}\left(PB\right){\left(PB\right)}^{T}E+\frac{1}{2}{\tilde{d}}^{2}+\frac{1}{2}{\hat{E}}^{T}\left(PB\right){\left(PB\right)}^{T}\hat{E}\\ & \;-{\hat{E}}^{T}\left(PB\right){\left(PB\right)}^{T}\hat{E}+\frac{1}{2}{\tilde{d}}^{2}-l\left(x\right){\tilde{d}}^{2}+\frac{{\tilde{d}}^{2}}{2}+\frac{l\left(x\right){∥\tilde{W}∥}^{2}h\left(x\right)}{2}-\frac{1}{2}\delta {∥\tilde{W}∥}^{2}\\ & \;+\frac{1}{2}\delta {∥W^{*}∥}^{2}+E^{T}PB\omega +{\hat{E}}^{T}PB\omega \\ \; & =-\frac{1}{2}E^{T}\left[Q-\left(PB\right){\left(PB\right)}^{T}\right]E-\frac{1}{2}{\hat{E}}^{T}\left(PB\right){\left(PB\right)}^{T}\hat{E}-\left[l\left(x\right)-\frac{3}{2}\right]{\tilde{d}}^{2}\\ & \;-\frac{\delta -l(x)h(x)}{2}{∥\tilde{W}∥}^{2}+\frac{1}{2}\delta {∥W^{*}∥}^{2}+\frac{1}{2}d_{m}^{2}+E^{T}PB\omega +{\hat{E}}^{T}PB\omega \end{array}$$

To further demonstrate system stability, these parameters can be designed to satisfy the following conditions:

(41) $$Q-\left(PB\right){\left(PB\right)}^{T}\geq 0$$

(42) $$l\left(x\right)-\frac{3}{2}\geq 0$$

(43) δ−l(x)h(x)≥0

Since the approximation error can be minimized sufficiently by designing the RBFNN.

Then, by enlarging the right side of inequality (40), to be exactly, the first four terms, we can establish

(44) $${\dot{V}}_{2}\leq kV_{2}+C$$

With

(45) $$k=\min(l\left(x\right)-\frac{3}{2},\delta -l\left(x\right)h\left(x\right),\lambda_{\min}\left(PBB^{T}P^{T}\right),1)$$

(46) $$C=\frac{d_{m}^{2}}{2}+\frac{\delta {∥W^{*}∥}^{2}}{2}+E^{T}PB\omega +{\hat{E}}^{T}PB\omega$$

By a similar way to solve a differential equation, inequality (44) can be mathematically “solved” to be

(47) $$V_{2}\leq \left(V_{2}{|}_{t=0}-\frac{C}{k}\right)e^{-kt}+\frac{C}{k}$$

where *t* denotes time. It is apparent that the right side of inequality (47) exponentially converge to $\frac{C}{k}$, proving $V_{2}$ to be bounded, therefore *E*, $\hat{E}$, $\tilde{d}$ and $\hat{W}$ are bounded. Thus, the system outputs can track their references asymptotically as in $\lim_{t\to \infty }x_{1}=\gamma_{d}$. This completes the proof. □

**Remark** **4.** *Unlike Z. Chen [31], W. Hao [32], X. Zhang [33], the DOB proposed in this paper does not use an RBF neural network to identify the inverse model of FJRs, but rather combines the nominal model with the RBF neural network estimation part to estimate the external disturbance. Compared with the methods of C. Liu [34], F. Xu [35], D. Shang [36] that only use RBFNN to tackle system uncertainties, disturbances and other factors, the controller proposed in this paper uses the estimation of the unknown term of the RBF neural network. In this paper, the nominal model is used in combination with the estimated part of RBF, rather than using the RBF neural network to identify the inverse model of Flexible-Joint Robots (FJRs).*

To elaborate, this design fundamentally reduces computational burden through pre-compensation of known information, with its theoretical principle as follows: Let the system model (8) be $f\left(x\right)=f_{\mathrm{known}}\left(x\right)+f_{\mathrm{unknown}}\left(x\right)$, where $f_{\mathrm{known}}\left(x\right)$ represents the system known part, which can be directly derived via the system’s nominal model $f_{0}\left(x\right)$; $f_{\mathrm{unknown}}\left(x\right)$ denotes the unknown part Δ, to be compensated by the proposed RBF neural network. Compared with traditional RBF neural network methods, the complexity of the unknown term Δ is significantly lower, thus enabling a reduction in the number of required neurons. This allows for effective reduction in computational load while achieving equivalent or even better control performance.

**Remark** **5.** *The error $\hat{E}$ between the actual outputs of the FJR and its estimated state outputs (20) is defined as*

$$\hat{E}={\left[\begin{array}{cccc} x_{1} & x_{2} & x_{3} & x_{4} \end{array}\right]}^{T}-{\left[\begin{array}{cccc} {\hat{x}}_{1} & {\hat{x}}_{2} & {\hat{x}}_{3} & {\hat{x}}_{4} \end{array}\right]}^{T}$$

*where ${\hat{x}}_{1},{\hat{x}}_{2},{\hat{x}}_{3},{\hat{x}}_{4}$ denote the outputs of the FJR Gray-Box State Space Model reconstructed using system prior knowledge, DOB estimate $\hat{d}$, and RBFNN estimate $\hat{\Delta }$. This model follows the state equation*

$$\dot{\hat{x}}=\left[\begin{array}{cccc} 0 & 1 & 0 & 0\\ 0 & 0 & 1 & 0\\ 0 & 0 & 0 & 1\\ 0 & 0 & 0 & 0 \end{array}\right]\hat{x}+\left[\begin{array}{c} 0\\ 0\\ 0\\ 1 \end{array}\right]\left(f_{0}\left(x\right)+g\left(x\right)u+\hat{d}+\hat{\Delta }\right)$$

*integrating nominal dynamics with uncertainty approximations.*

**Figure 4:**
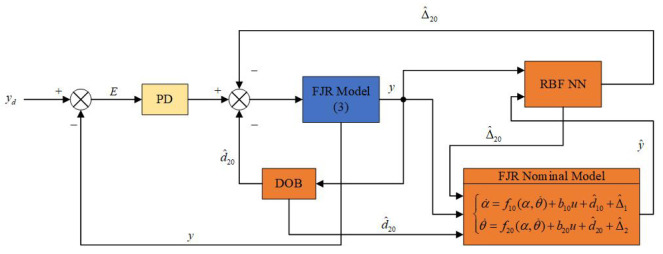
Block diagram of DOB control based on RBF neural network.

The control algorithm principle block diagram based on neural network DOB is shown in Figure 4.

## 4. Simulation and Experimental Results

In this section, the proposed controller is implemented on an underactuated FJR to validate its effectiveness and robustness.

To verify the proposed controller, state feedback control, RBF neural network adaptive control, and state feedback control based on disturbance observer are used for comparison. However, in practical experiments, the FJR cannot directly measure $x_{3}$ and $x_{4}$. Given this limitation, Equation (7) will be used for research in the experiment. In state feedback control, the state value represents the error between the output value and the target value. It can be observed that the form of state feedback control used in this paper is similar to PD control, which will be represented by PD control hereinafter.

As the PD control, the control law of the algorithm for system (7) should be designed as follows:

(48) $$u=\frac{1}{b_{20}\left(z\right)}\left[-f_{20}\left(z\right)+K^{T}E+{\ddot{z}}_{3d}\right]$$

To ensure the stability of the system, we need to artificially design a suitable PD controller parameter *K*.

The second algorithm is RBF neural network adaptive control, which uses RBF neural network to estimate the overall dynamic model of flexible joint manipulator system and combines PD controller. The performance of RBF neural network is affected by the center vector c and base width b, which determine the fitting accuracy and generalization ability of the network. Its control law is:

(49) $$\begin{array}{c} u=\frac{1}{b_{20}\left(z\right)}\left[{\hat{\Delta }}_{f_{2}}+K^{T}E+{\ddot{z}}_{3d}\right] \end{array}$$

where ${\hat{\Delta }}_{f_{2}}={W_{1}}^{T}h\left(x\right)$ Is the estimate of the system dynamics model by RBFNN. According to the above proof, the weight update rate can also be obtained as: ${\dot{\hat{W}}}_{1}=-\mu_{2}E^{T}PBh\left(x\right)$.

The third algorithm is PD control based on DOB [37]. The DOB can be designed according to the known nominal model of the flexible joint manipulator, so the corresponding disturbance estimate can be obtained according to (18). Note that the performance of the DOB is influenced not only by the accuracy of the nominal model but also by the gain l(x); generally, a larger gain leads to a faster response. Additionally, since $b_{1}=-b_{2}$ in (1) and (2), it is sufficient to design a DOB for either (1) or (2) and compensate for the estimated disturbance, thereby eliminating the influence of disturbances in the FJR. Thus, the corresponding controller is designed as follows:

(50) $$\begin{array}{c} u=\frac{1}{b_{20}\left(z\right)}\left[-f_{20}\left(z\right)-d_{20}+K^{T}E+{\ddot{z}}_{3d}\right] \end{array}$$

Since all experimental comparison schemes include both a PD module and a DOB module, to ensure a fair comparison among the four schemes, the parameters of the shared PD controller are uniformly adopted for all four controllers as follows: $K={\left[\begin{array}{ccc} Kp_{1} & Kd_{1} & \begin{array}{cc} Kp_{2} & Kd_{2} \end{array} \end{array}\right]}^{T}$. Meanwhile, both the DOB-based PD control and the controller proposed in this paper incorporate a DOB. Therefore, the two designed DOB algorithms are kept consistent, and the same gain value l(x) is used for both.

### 4.1. Simulation Results

To clearly validate the external disturbance suppression capability of the proposed controller, the verification scheme from the previous section is adopted with fixed controller parameters, consistently applied in both simulation and experimental verifications.

**Figure 5:**
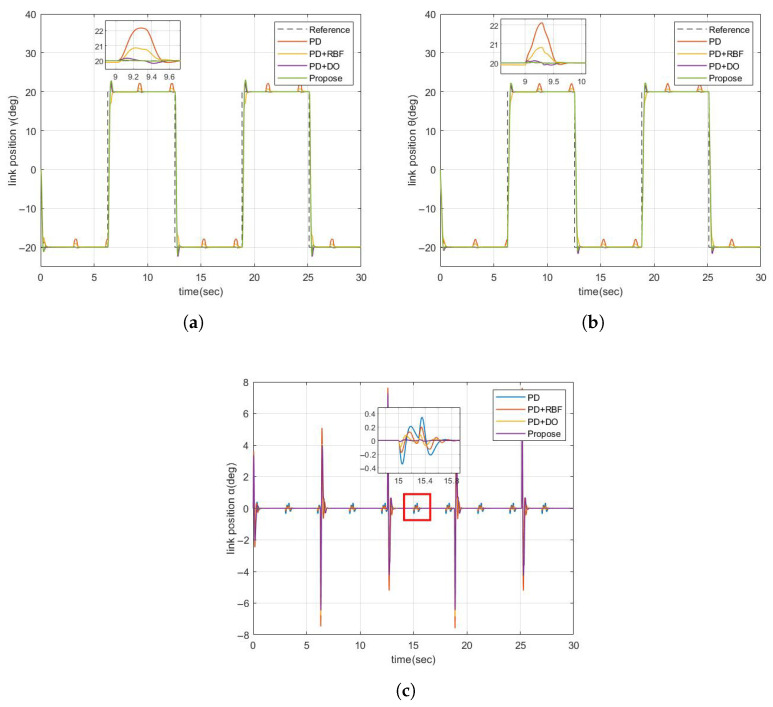
Comparison diagram of the tracking response of FJR under the disturbance of pulse signals. (**a**) Comparison diagram of the tracking response of FJR for γ; (**b**) Comparison diagram of the tracking response of FJR for θ; (**c**) Comparison diagram of the tracking response of FJR for α.

The reference position signal is a square wave signal with the amplitude of 20 deg and the period of 0.5 hz. In order to verify the anti-disturbance ability, a PWM wave signal with an amplitude of 20 deg is artificially added as an external disturbance. As can be seen in Figure 5a,b, when an external disturbance occurs, the proposed controller can effectively suppress the disturbance, which has obvious advantages over the other three controllers.

### 4.2. Experimental Results

Based on the parameters of the Quanser FJR given in Table 1, a corresponding dynamic model can be calculated. However, in experiments, factors such as motor friction, sampling errors, load disturbances, etc. will cause the calculated dynamic model to differ from the actual dynamic model. In this subsection, the results of the following four experiments will be shown.

**Figure 6:**
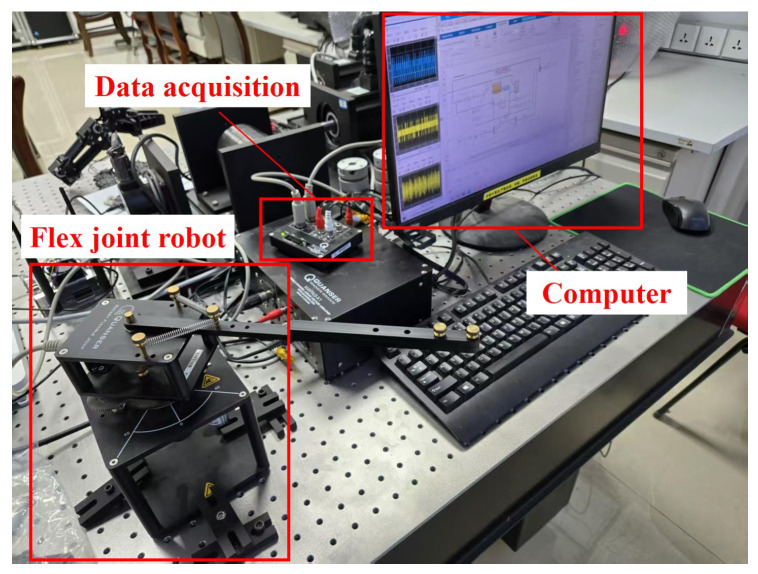
FJR Experimental Setup.

To verify the correctness of the simulation results in this paper, the proposed control scheme was implemented on the FJR test platform, as shown in Figure 6. Figure 6 shows that the experimental setup consists of the FJR, data processing equipment, and a computer.

#### 4.2.1. Verifying the Sine Tracking Performance

**Figure 7:**
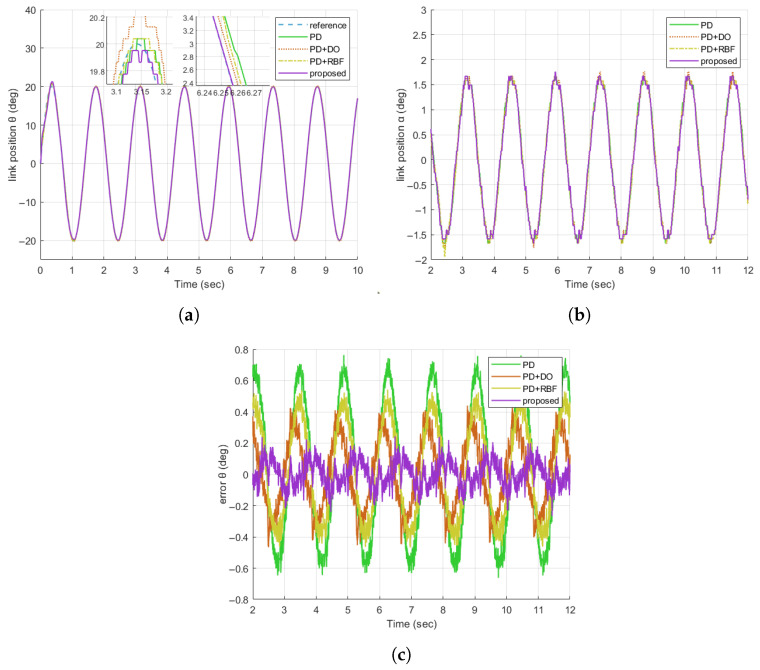
Comparison of tracking responses of FJR without disturbance. (**a**) Comparison diagram of the tracking responses of θ; (**b**) Comparison diagram of the tracking responses of α; (**c**) Comparison diagram of the tracking errors of θ.

In Experiment 1, we verify the tracking performance of the Flexible Joint Robot (FJR) system under nominal operating conditions (free from human-induced external interference). A sinusoidal reference signal with an amplitude of 20 deg and a frequency of 4.5 rad/s is employed. Figure 7a–c present the motor position, connecting rod position, and motor tracking error of the four algorithms, respectively. The connecting rod position results depicted in Figure 7a illustrate that the proposed method (represented by the purple curve) most closely aligns with the reference sinusoidal signal, achieving a steady-state error of less than 0.2 deg. This outperforms other comparative schemes and serves to confirm its superior capability in compensating for inherent system dynamics, such as frictional effects. The motor tracking error results in Figure 7c quantitatively substantiate these findings: the proposed method achieves a root-mean-square error (RMSE) of less than 0.0831 deg, outperforming other schemes with lower overall error magnitude and greater consistency throughout the entire operation. Overall, these results demonstrate that the proposed control method can effectively compensate for uncertainties and estimation errors, yielding enhanced control precision in the steady-state regime.

#### 4.2.2. Verification of Anti-Disturbance Ability

To verify the applicability of the proposed control strategy under different disturbance scenarios, two sets of validation experiments are presented below: one involves sinusoidal disturbances, simulating periodic external interference; the other focuses on complex disturbances, comprehensively considering the effects of non-periodic and abrupt interference.

**Figure 8:**
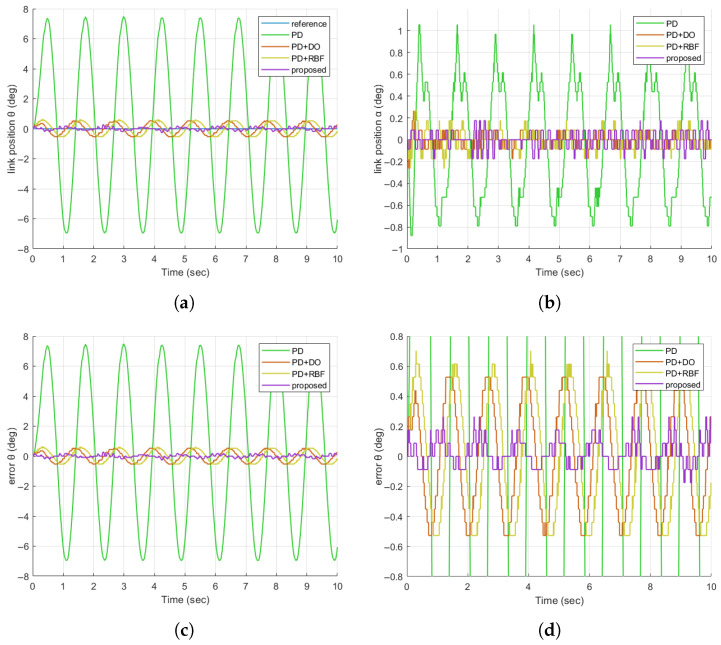
Comparison of FJR tracking responses in the case of sinusoidal disturbed signals. (**a**) Comparison diagram of the tracking responses of θ; (**b**) Comparison diagram of the tracking responses of α; (**c**) Comparison diagram of the tracking errors of θ; (**d**) Enlarged comparison diagram of the tracking errors of θ.

A. In Experiment 2, we evaluate the disturbance rejection capability of the proposed controller for the FJR system, focusing on its ability to counter external vibrations—a critical challenge in practical robotic applications (e.g., operation near machinery). A sinusoidal disturbance signal (3 deg amplitude, 5 rad/s frequency) is superimposed on the control input to simulate such vibrations, which typically displace the link from its equilibrium position. The performance of the proposed method, alongside three comparative schemes, is analyzed using the results presented in Figure 8a–d.

The link position curves in Figure 8a show that the proposed controller (purple) maintains the smallest deviation from the reference sinusoid during disturbance. For example, at 2–4 s (peak disturbance influence), the PD+DO method (orange) exhibits deviations of 1.2 deg, while the proposed method limits deviations to <0.3 deg. This advantage stems from its real-time disturbance estimation (unlike PD/PD+DO, which rely on fixed gain compensation) and adaptive error correction (surpassing PD+RBF in dynamic disturbance scenarios). For practical FJR applications, this translates to higher task accuracy (e.g., precision grasping under vibrations) and reduced mechanical wear, addressing key limitations of comparative methods.

**Figure 9:**
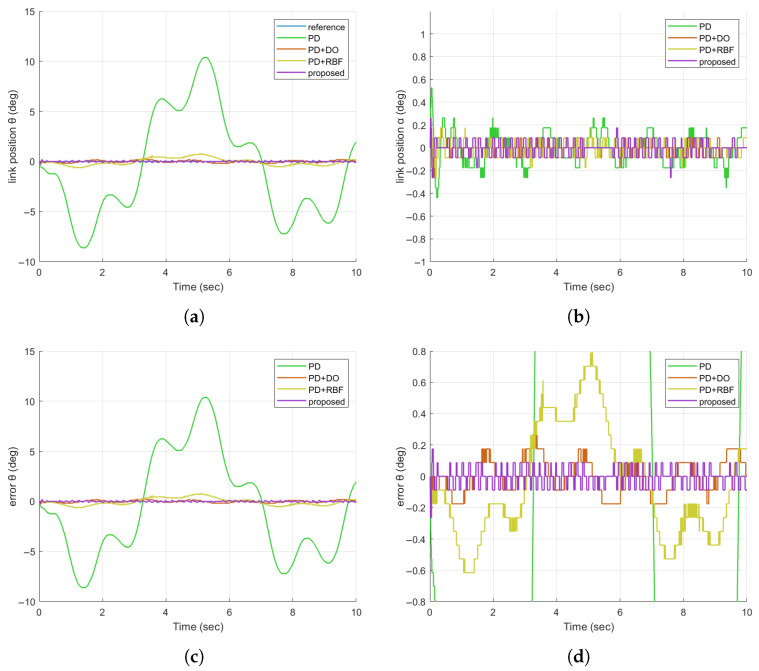
Comparison of FJR tracking responses in the case of multi-frequency composite disturbance signals. (**a**) Comparison diagram of the tracking responses of θ; (**b**) Comparison diagram of the tracking responses of α; (**c**) Comparison diagram of the tracking errors of θ; (**d**) Enlarged comparison diagram of the tracking errors of θ.

In Experiment 3, we assess the disturbance rejection capability of the proposed controller for the FJR system against more complex disturbance patterns, which are common in real-world environments where multiple interference sources coexist (e.g., robotic operations near mixed industrial machinery). A multi-frequency composite disturbance signal—combining two sinusoidal waves of different frequencies and a triangular wave—is imposed on the control input to simulate such intricate vibrations, which tend to cause persistent and irregular displacement of the link from its equilibrium position. The performance of the proposed method, in comparison with the three reference schemes, is analyzed based on the results depicted in Figure 9a–d.

The motor position trajectories in Figure 9a demonstrate that the proposed controller (purple) maintains the most stable tracking performance amid the multi-component disturbance. Notably, during the interval of 4–6 s—where the triangular wave introduces abrupt amplitude changes and the two sinusoidal waves create beat-like oscillations—the PD+RBF method (yellow) exhibits maximum deviations of 0.8 deg, while the proposed method constrains deviations to <0.087 deg. Quantitative results in Figure 9c reinforce this—the proposed method achieves an RMSE of 0.0605.

For practical FJR applications involving diverse interference sources (e.g., factory floors with overlapping machinery vibrations), this capability ensures reliable task execution under complex disturbances.

#### 4.2.3. Verifying the Robustness

**Figure 10:**
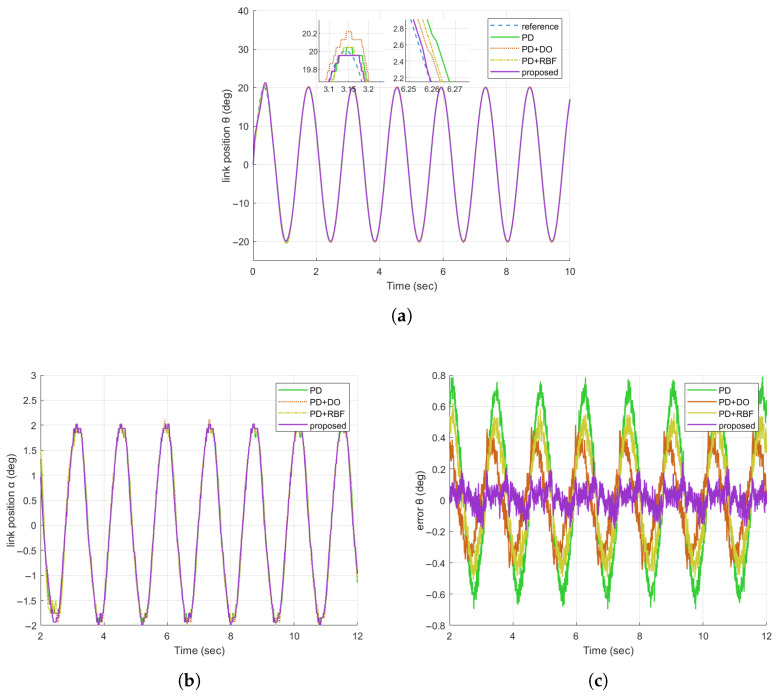
Tracking response comparison of the FJR with payload and no disturbance. (**a**) Comparison diagram of the tracking responses of θ; (**b**) Comparison diagram of the tracking responses of α; (**c**) Comparison diagram of the tracking errors of θ.

**Figure 11:**
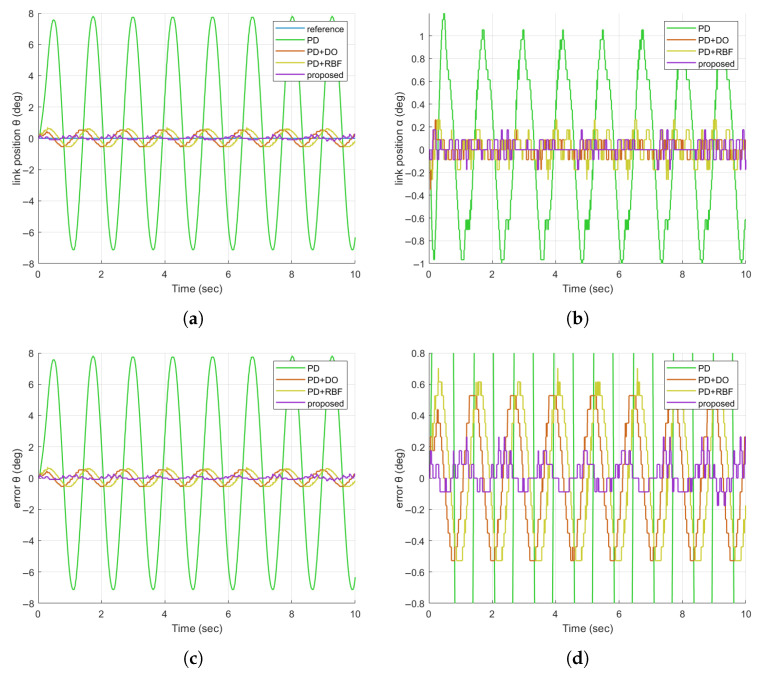
Tracking response comparison of the FJR with payload and disturbance. (**a**) Comparison diagram of the tracking responses of θ; (**b**) Comparison diagram of the tracking responses of α; (**c**) Comparison diagram of the tracking errors of θ; (**d**) Enlarged comparison diagram of the tracking errors of θ.

**Figure 12:**
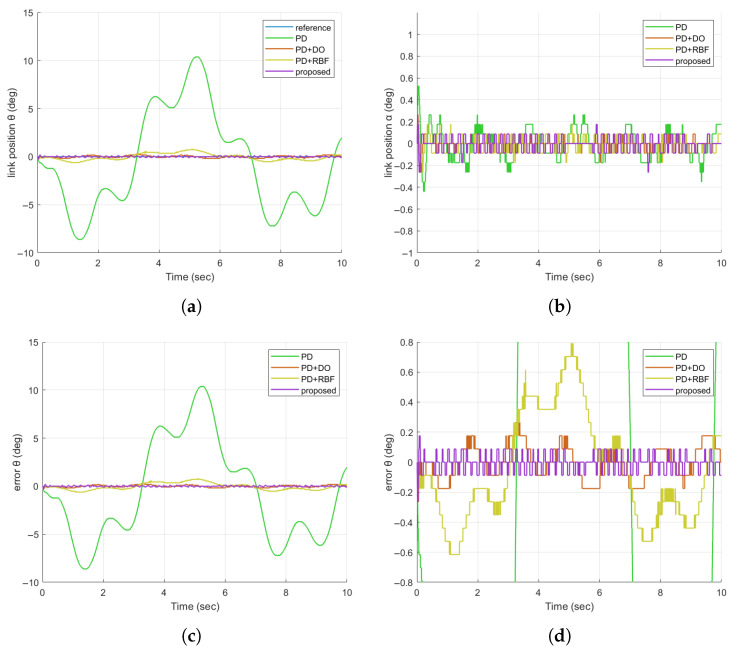
Tracking response comparison of the FJR with payload and composite disturbance. (**a**) Comparison diagram of the tracking responses of θ; (**b**) Comparison diagram of the tracking responses of α; (**c**) Comparison diagram of the tracking errors of θ; (**d**) Enlarged comparison diagram of the tracking errors of θ.

In experiment 4, we increased the inertia at the load end of the flexible joint manipulator, resulting in a change in the dynamics model of the system, which will further reduce the stability of the connecting rod end and make it more susceptible to disturbance. Under this condition, we repeat the steps of experiment 1 and experiment 2. As can be seen from Figure 10a–c, even if the load inertia is changed, the proposed algorithm can also achieve relatively accurate position tracking. Figure 11a–d and Figure 12 also clearly shows that our proposed algorithm effectively reduces the influence caused by the increase of inertia, so that the system still has a strong anti-disturbance ability.

## 5. Conclusions

This paper proposes a Radial Basis Function neural network disturbance observer (RBFNNDOB)-based state feedback controller for flexible joint robots (FJR), which exhibit inherent uncertainties, nonlinear dynamics, and susceptibility to external disturbances. The integration of DOB and RBFNN effectively mitigates the effects of system dynamic uncertainties and external disturbances. Additionally, by fully utilizing the known parameters of the FJR, this approach reduces the controller’s computational load while enhancing control accuracy and response speed. The system stability was proved via the Lyapunov method. The proposed controller was validated through simulations and experiments on a FJR. Comparative experimental results demonstrate that the proposed controller exhibits superior tracking accuracy and enhanced disturbance rejection capabilities.

Although experimental results demonstrate the advantages of the proposed method, significant room for improvement remains. For instance, future research could focus on optimizing the neural network structure to enhance both accuracy and computational efficiency. Moreover, it is crucial to address constraints on the system input: as highlighted by the observation that the two rectilinear springs attached to the link impose restrictions on the link rotation angle—an aspect not fully considered in the current study—and further, the practical limits of input torque, which can lead to saturation under extreme disturbance conditions and compromise control performance. Therefore, subsequent work will prioritize investigating the impact of such input limitations by exploring adaptive control strategies that account for both rotational angle constraints and torque boundaries, aiming to ensure stable and efficient system operation even under input constraints. This will not only boost the practical applicability of the proposed method but also deepen understanding of the system’s performance boundaries.

## Figures and Tables

**Table 1:** Nominal parameters of the FJR manipulator.

Parameter	Meaning	Value
$R_{m}$	Armature Resistance (Ω)	2.6
$K_{m}$	Motor Back-EMF Constant (V·s/rad)	0.00767
$K_{t}$	Motor Torque Constant (N·m/A)	0.00767
$J_{\mathrm{arm}}$	Total Arm Inertia (kg·m^2^)	0.0019
$J_{\mathrm{eq}}$	Equivalent Inertia (kg·m^2^)	0.0021
$K_{g}$	High Gear Ratio	70:1
$K_{s}$	Joint Stiffness (N·m/rad)	1.2485
$B_{\mathrm{eq}}$	Equivalent Viscous Damping (N·m·s/rad)	0.004
$\eta_{g}$	Gearbox Efficiency	0.9
$\eta_{m}$	Motor Efficiency	0.69

## Data Availability

Data is contained within the article.
